# The Prognostic Significance of PDE7B in Cytogenetically Normal Acute Myeloid Leukemia

**DOI:** 10.1038/s41598-019-53563-x

**Published:** 2019-11-18

**Authors:** Ling Cao, Weilong Zhang, Xiaoni Liu, Ping Yang, Jing Wang, Kai Hu, Xiuru Zhang, Weiyou Liu, Xue He, Hongmei Jing, Xiaoliang Yuan

**Affiliations:** 1 Gannan Medical University, Ganzhou, 341000 China; 2 Department of Hematology, Lymphoma Research Center, Peking University Third Hospital, Beijing, 100191 China; 3 Department of Respiratory Medicine, The First Affiliated Hospital of Gannan Medical University, Ganzhou, 341000 China; 4 Department of Pathology, Beijing Tiantan Hospital Affiliated with Capital Medical University, No. 6 Tiantan Xili, Beijing, 100050 China

**Keywords:** Microarray analysis, Microarray analysis, Microarray analysis, Microarray analysis, Myeloma

## Abstract

Acute myeloid leukemia (AML) is a malignant hematological disease in which nearly half have normal cytogenetics. We have tried to find some significant molecular markers for this part of the cytogenetic normal AML, which hopes to provide a benefit for the diagnosis, molecular typing and prognosis prediction of AML patients. In the present study, we calculated and compared the gene expression profiles of cytogenetically normal acute myeloid leukemia (CN-AML) patients in database of The Cancer Genome Atlas (TCGA), Gene Expression Omnibus (GEO) and dataset Vizome (a total of 632 CN-AML samples), and we have demonstrated a correlation between PDE7B gene and CN-AML. Then we proceeded to a survival analysis and prognostic risk analysis between the expression levels of PDE7B gene and CN-AML patients. The result showed that the event-free survival (EFS) and overall survival (OS) were significantly shorter in CN-AML patients with high PDE7B levels in each dataset. And we detected a significantly higher expression level of PDE7B in the leukemia stem cell (LSC) positive group. The Cox proportional hazards regression model showed that PDE7B is an independent risk predictor for CN-AML. All results indicate that PDE7B is an unfavorable prognostic factor for CN-AML.

## Introduction

Acute myeloid leukemia (AML) is a group of malignant clonal hematological diseases that originate from hematopoietic stem cells (HSC) and is highly heterogeneous^1–4^. In 1976, the French-America-Britain (FAB) classification based on traditional bone marrow cell morphology and cytochemical staining divided AML into 8 subtypes of M0 ~ M7^5^. However, the classification criteria have certain limitations on the role of clinical prognosis and guiding treatment. In 2001, the international hematology community launched the WHO diagnostic classification system for hematopoietic malignancies, namely the MICM (Morphological, Immunological, Cytogenetics, Molecular Biology) standard^6^. This standard further systematically and comprehensively classified AML, which has important guiding significance for clinical treatment.

In recent years, the role of gene mutations in the pathogenesis of AML has been increasingly revealed, which has become a new marker of molecular diagnosis and a potential therapeutic target. In 2013, it has been reported that after the genome-wide or whole-exome sequencing analysis of 200 adult patients of de novo AML, 23 genes (including DNMT3A, FLT3, NPM1, IDH1, IDH2, CEBPA, U2AF1, EZH2, SMC1A, SMC3, etc.) with a higher-than-expected mutation prevalence were summarized, and 57 novel fusion genes were identified^7^. Mutant genes obtained from 200 samples can be classified into a total of 9 categories according to biological function definitions, including transcription-factor fusions genes (The most statistically significant set, PML-RARA, MYH11-CBFB, RUNX1-RUNX1T1, PICALM-MLLT10, NUP98-NSD1, etc.), the gene encoding nucleophosmin (NPM1), tumor-suppressor genes (TP53, WT1, PHF6, etc.), DNA-methylation-related genes (DNMT3A, DNMT3B, DNMT1, TET1, TET2, IDH1, IDH2, etc.), signal transduction pathway-related genes (FLT3, KIT, KRAS, NRAS, PTPs, Ser-Thr kinase, etc.), chromatin-modifying genes (ASXL1, EZH2, KDM6A, MLL-related fusion genes, MLL-PTD, NUP98-NSD1, etc.), myeloid transcription-factor genes (RUNX1, CEBPA, etc.), cohesin-complex genes (STAG2, RAD21, etc.), and spliceosome-complex genes (SRSF2, SF3B1, ZRSR2, U2AF1, etc.). The discovery of these genes makes an important contribution to the accurate diagnosis of AML.

With the continuous development of precision medicine, it has enriched the treatment of AML. However, even with high-dose combination chemotherapy and hematopoietic stem cell transplantation, the recurrence rate of AML is still high and the 5-year overall survival rate is less than 40%^8^. Although cytogenetic examination is important for patient outcomes and the choice of individualized treatment regimens^9^, while nearly half of AML patients lack clonal chromosomal abnormalities^10^ and it may not be effective for this subset of patients. Therefore, it is necessary to find more potential biomarkers for prognosis prediction and treatment of AML patients with normal cytogenetic.

PDE7B is a phosphodiesterase (PDE) with high affinity and specificity for cAMP (cyclic adenosine monophosphate), which reduces intracellular cAMP concentration by hydrolysis^11,12^. A number of studies have shown that PDE7B may play a role in a variety of central nervous system diseases by affecting cAMP levels, and found that PDE7B may be involved in dopaminergic signaling in striatal neurons and play a key role in memory function^11,13–15^. PDE7B also has been found to have prognostic significance in a variety of cancers, such as chronic lymphocytic leukemia (CLL, high expression level of PDE7B acts as a bad prognostic factor)^16^, mantle cell lymphoma (high expression level of PDE7B is a poor prognostic indicator)^17^, glioblastoma (high expression level of PDE7B has a worse prognosis)^18^ and breast cancer (high expression level of PDE7B predicts poor prognosis)^19^. In addition, PDE7B is overexpressed in glioblastoma and serves as an important cell growth medium^18^. cAMP promotes growth arrest and/or apoptosis of various types of lymphoma, particularly CLL, and PDE7 inhibitors can increase cAMP concentration and kill CLL cells^20,21^, which is suggesting that PDE7B may act as a therapeutic target in CLL. Intracellular cAMP levels are critical for the differentiation of leukemia cells^22^, which shows the potential of PDE7B as a cAMP-specific hydrolase in the prognostic significance and treatment of leukemia, including AML.

However, there have been no reports to date on the expression of PDE7B in AML patients and its relationship with the prognosis of AML patients. Therefore, we investigated the expression level of PDE7B in CN-AML patients and systematically analyzed its relationship with the prognosis of CN-AML patients. As the results presented in our research, we found that CN-AML patients with high expression of PDE7B had shorter survival times, suggesting that the PDE7B gene is an unfavorable prognostic marker for CN-AML.

## Results

### High expression level of PDE7B is a poor prognostic marker for CN-AML patients

**Figure 1:**
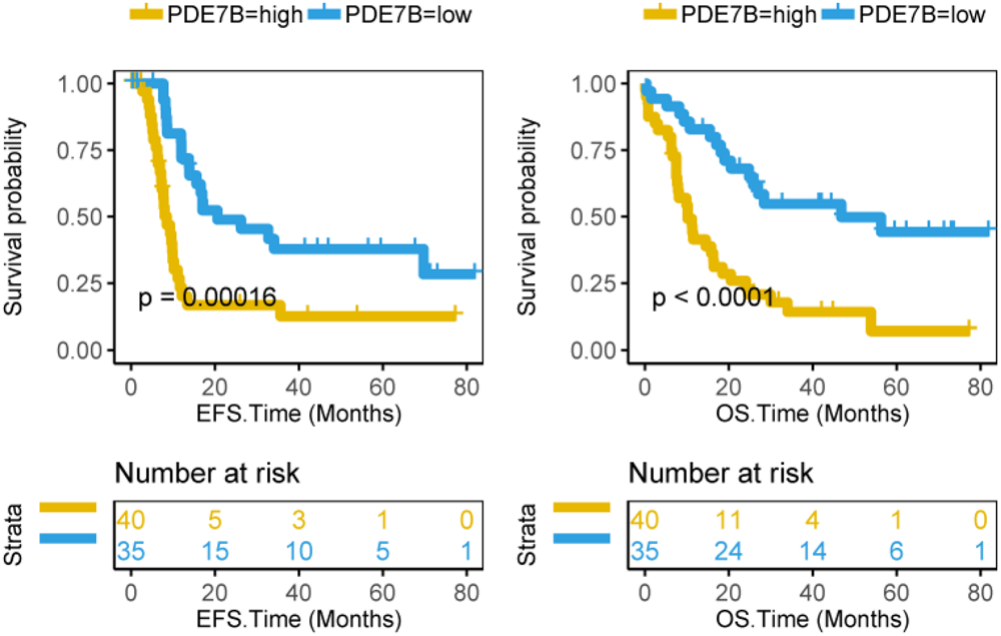
The survival between the PDE7B high group and the PDE7B low group of CN-AML patients are compared. Survival analysis of 75 CN-AML patients in the TCGA database, event-free survival time (EFS, months) on the left and overall survival time (OS, months) on the right. The survival curve is compared with the log-rank test. The x-axis represents the survival time and the y-axis represents the survival probability.

**Figure 2:**
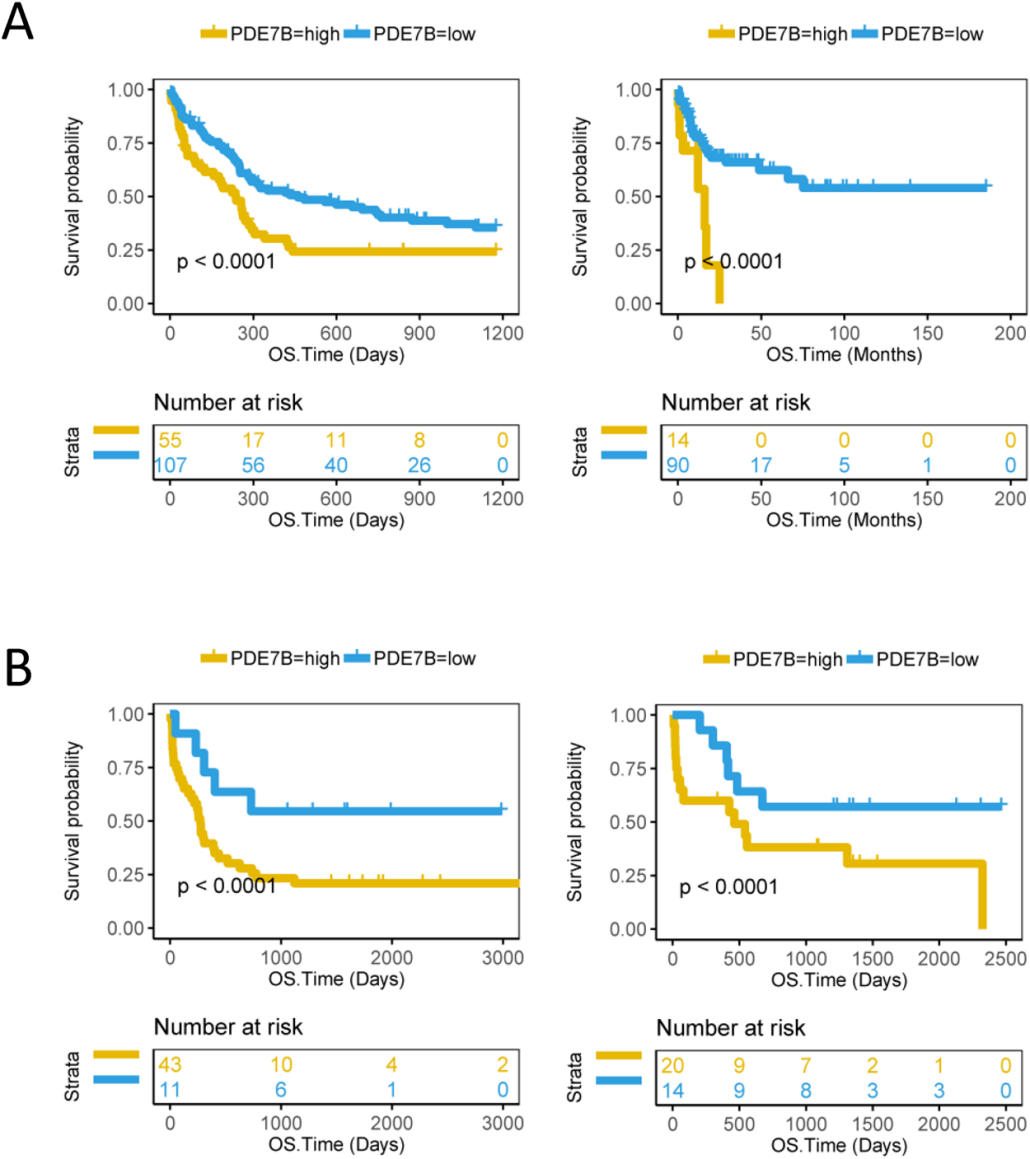
Compare the survival between the PDE7B high group and the PDE7B low group of CN-AML patients from different datasets. (**A**) The left side is the overall survival time (OS, days) of 162 CN-AML patients in the GSE12417 U133B dataset and the right side is the OS (months) of 104 CN-AML patients in the GSE71014 dataset. (**B**) On the left is the OS time (days) of 54 CN-AML patients in the GSE22778 GPL8653 dataset and on the right is the OS time (days) of 34 CN-AML patients in the GSE22778 GPL10107 dataset. The survival curve is compared using the log-rank test. The x-axis represents the OS time and the y-axis represents the survival probability.

We performed a survival analysis of 75 CN-AML patients from the TCGA database and found that EFS (P = 0.00016, Log-rank test, Fig. 1) and OS (P < 0.0001, Log-rank test, Fig. 1) were significantly lower in the high expression level of PDE7B group than in the low level PDE7B group. The expression levels of PDE7B between the high group and low group was shown (Supplementary Fig. 1). We also analyzed the OS time of CN-AML patients from several subsets of another database GSE. The results showed that the OS time of the low expression level of PDE7B group in the datasets GSE12417 U133B (162 patients, P < 0.0001, Log-rank test, Fig. 2A left), GSE71014 (104 patients, P < 0.0001, Log-rank test, Fig. 2A right), GSE22778 GPL8653 (54 patients, P < 0.0001, Log-rank test, Fig. 2B left) and GSE22778 GPL10107 (34 patients, P < 0.0001, Log-rank test, Fig. 2B right) was significantly longer. The expression levels of PDE7B for these datasets were shown (Supplementary Fig. 2A,B). We also validated the data from a recently published study and obtained similar results. The OS time of 396 AML patients (P < 0.0001, Log-rank test, Supplementary Fig. 3A left) and 203 CN-AML patients (P < 0.0001, Log-rank test, Supplementary Fig. 3A right) with high level PDE7B was significantly lower than that of low level PDE7B. And the expression level of PDE7B was shown (396 AML samples on the left and 203 CN-AML samples on the right, Supplementary Fig. 3B).

### Prognostic significance of PDE7B level in AML patients after treatment

**Figure 3:**
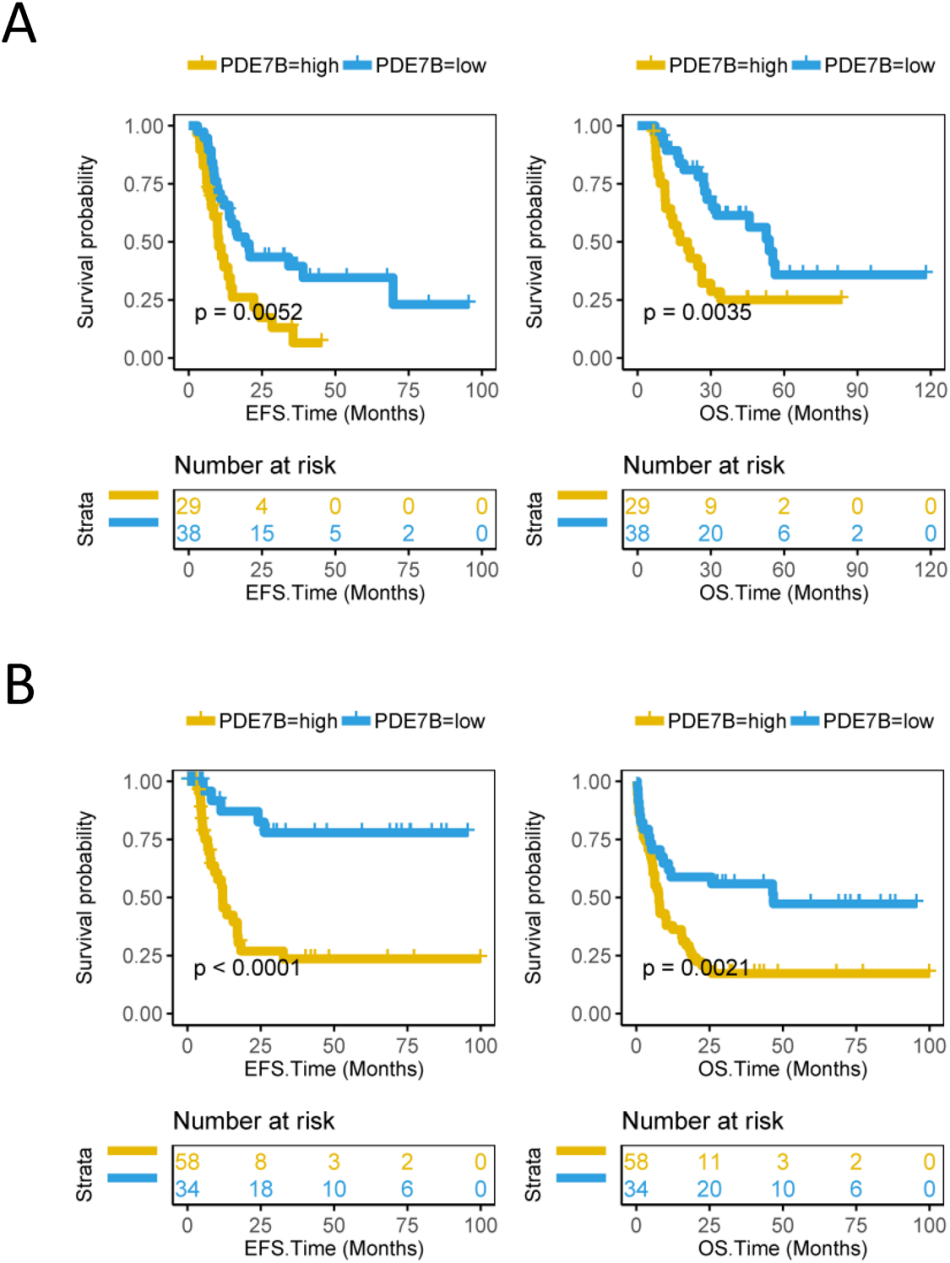
The survival between PDE7B high group and PDE7B low group of AML patients who had received Allo-HSCT or chemotherapy are compared. (**A**) Survival analysis of 67 AML patients who had received Allo-HSCT in the TCGA database, event-free survival time (EFS, months) on the left and overall survival time (OS, months) on the right. (**B**) Survival analysis of 92 AML patients who received chemotherapy in the TCGA database, EFS time (months) on the left and OS time (months) on the right. The survival curve is compared with the log-rank test. The x-axis represents the survival time and the y-axis represents the survival probability.

In the TCGA database, 67 AML patients who had received Allo-HSCT were included. The result was that a significant prolongation of the survival time in AML patients who had received Allo-HSCT with low expression level of PDE7B relative to AML patients with high level PDE7B (EFS: P = 0.0052, Log-rank test, Fig. 3A; OS: P = 0.0035, Log-rank test, Fig. 3A). We also tested another group of 92 AML patients who had received chemotherapy in the TCGA database, and the results were similar to the previous ones. The EFS time (P < 0.0001, Log-rank test, Fig. 3B) and OS time (P = 0.0021, Log-rank test, Fig. 3B) of AML patients who had received chemotherapy in the low expression level of PDE7B group were significantly longer than the high level PDE7B group. The expression levels of PDE7B between the high group and low group was also shown (Supplementary Fig. 2C).

### The relationship between the gene expression level of PDE7B and leukemia stem cells

**Figure 4:**
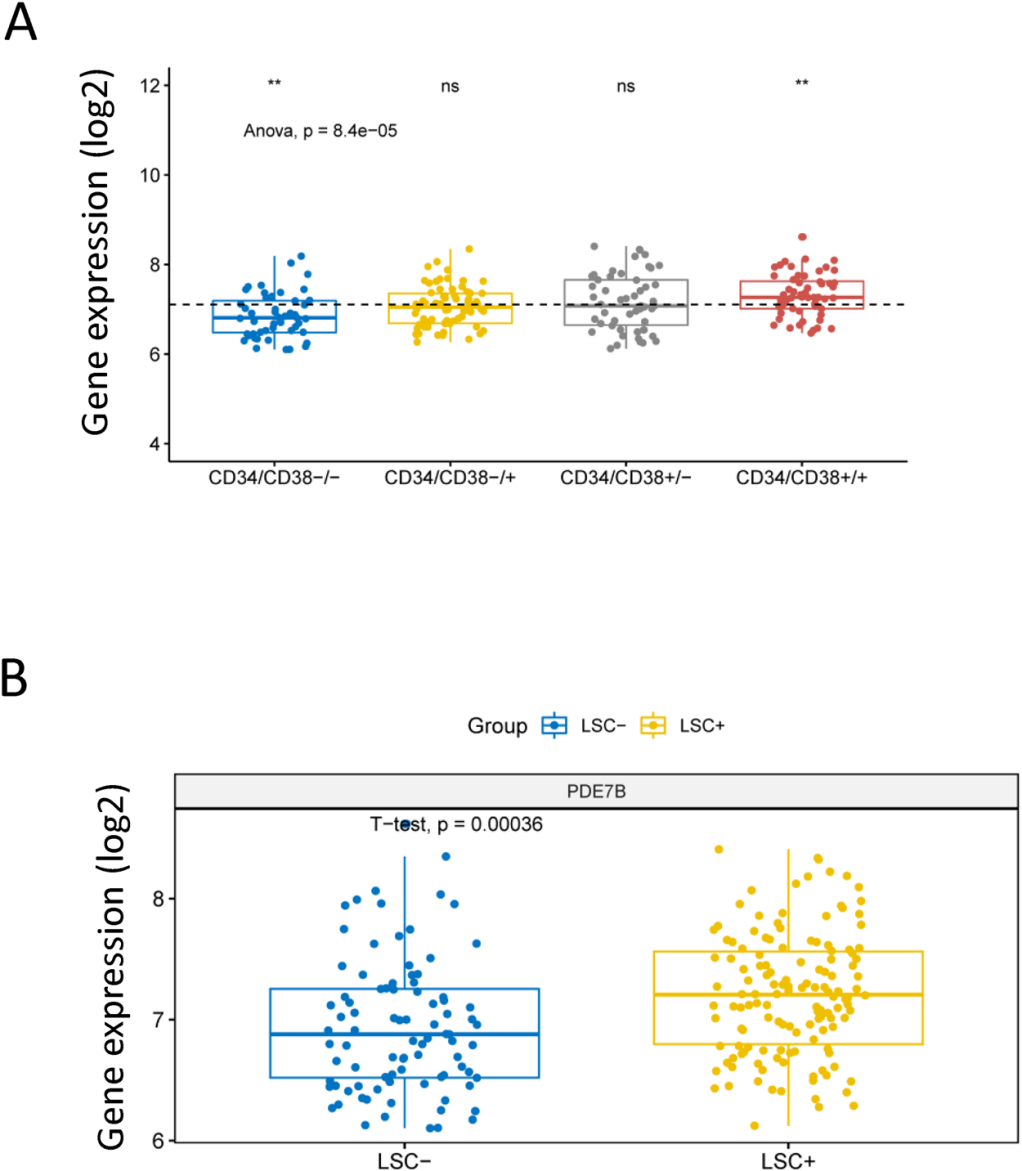
Compare the difference in expression levels of PDE7B in AML patients between different molecular types or leukemia stem cell positive/negative. (**A**) The 227 samples from 78 AML patients in the GSE76004 dataset are divided into CD34^−^/CD38^−^, CD34^−^/CD38^+^, CD34^+^/CD38^−^ and CD34^+^/CD38^+^, and the differences of PDE7B expression in the four groups are compared. ANOVA test is used for comparison between the four groups. ns, no significance, P > 0.05; **P < = 0.001. The dotted line represents the average level of PDE7B expression of all AML patients. The x-axis represents four molecular types, and the y-axis represents gene expression levels (log2). (**B**) The 227 samples are divided into leukemia stem cell (LSC) positive group and LSC negative group, and the difference in expression of PDE7B gene between the two groups is compared. The unpaired t test (two sided) is used for comparison between the two groups. The x-axis represents LSC positive/negative and the y-axis represents gene expression level (log2).

Leukemia stem cell (LSC) play an important role in the occurrence and development of AML, and CD34^+^ and CD38^−^ are the surface molecular phenotype of LSCs. To investigate the association between gene expression levels of PDE7B and leukemia stem cells, 227 samples from dataset GSE76004 were included in the study. As a result, the gene expression levels of PDE7B were significantly different in four different combinations based on CD34^+/−^ and CD38^+/−^ (P = 8.4e-05, ANOVA test, Fig. 4A). Specifically, the gene expression level of PDE7B which compared to the average level was significantly reduced in the CD34^−^/CD38^−^ group, and was significantly increased in the CD34^+^/CD38^+^ group, while there was no significant difference in CD34^−^/CD38^+^ group and CD34^+^/CD38^−^ group. The gene expression level of PDE7B in the LSC positive group was significantly higher than that in the LSC negative group (P = 0.00036, unpaired t test, two sided, Fig. 4B).

### Baseline characteristics of 75 CN-AML patients in the TCGA database according to the level of PDE7B

First, we divided 75 CN-AML patients from the TCGA database into PDE7B high group (40 patients) and PDE7B low group (35 patients) according to the expression level of PDE7B. Then, the difference of the baseline characteristics of CN-AML between the PDE7B high group and the PDE7B low group were analyzed. White blood cell (WBC) counts were significantly higher in the PDE7B high group than in the PDE7B low group (P = 0.008, unpaired t test, two sided, Supplementary Table 1). And the three groups (wild type, mutation, and unknown) of the IDH1 (isocitrate dehydrogenase 1) gene were significantly different between the PDE7B high group and the PDE7B low group (P = 0.01, Fisher’s exact test, Supplementary Table 1). We also investigated other AML-related mutant genes including DNMT3A (DNA methyltransferase 3A, P = 0.097, Fisher’s exact test, Supplementary Table 1), NPM1 (nucleophosmin 1, P = 0.158, Fisher’s exact test, Supplementary Table 1), TET2 (tet methylcytosine dioxygenase 2, P = 0.289, Fisher’s exact test, Supplementary Table 1), FLT3 (fms related tyrosine kinase 3, P = 0.059, Fisher’s exact test, Supplementary Table 1), IDH2 (isocitrate dehydrogenase 2, P = 0.5, Fisher’s exact test, Supplementary Table 1), RUNX1 (runt related transcription factor 1, P = 1, Fisher’s exact test, Supplementary Table 1), NRAS (NRAS proto-oncogene, P = 0.679, Fisher’s exact test, Supplementary Table 1), WT1 (Wilms tumor 1, P = 0.82, Fisher’s exact test, Supplementary Table 1), CEBPA (CCAAT enhancer binding protein alpha, P = 1, Fisher’s exact test, Supplementary Table 1), PTPN11 (protein tyrosine phosphatase, non-receptor type 11, P = 0.82, Fisher’s exact test, Supplementary Table 1), and KRAS (KRAS proto-onco gene, P = 1, Fisher’s exact test, Supplementary Table 1). As the results showed that they were not significant differences between the PDE7B high group and the PDE7B low group. The numerous treatment regimens (induction: P = 0.475, Fisher’s exact test, Supplementary Table 1; transplant: P = 0.717, Fisher’s exact test, Supplementary Table 1; before transplant: P = 0.719, Fisher’s exact test, Supplementary Table 1) and relapse (P = 0.473, Fisher’s exact test, Supplementary Table 1) of AML were not significantly associated with PDE7B expression levels. Others, such as sex (P = 0.646, Fisher’s exact test, Supplementary Table 1), age (P = 0.131, unpaired t test, two sided, Supplementary Table 1), race (P = 0.353, Fisher’s exact test, Supplementary Table 1), FAB subtypes (P = 0.541, Fisher’s exact test, Supplementary Table 1), bone marrow blast (BM-BLAST, P = 0.937, unpaired t test, two sided, Supplementary Table 1), and peripheral blood blast (PB-BLAST, P = 0.461, unpaired t test, two sided, Supplementary Table 1) were also did not differ significantly between the PDE7B high group and the PDE7B low group.

### Multivariate analysis of CN-AML patients from TCGA database

We performed a multivariate analysis of the 75 CN-AML patients from the TCGA database. By comparing the EFS (HR: 4.8206, 95% CI: 1.9815–11.7273, P = 0.0005, Cox regression analysis, Table 1) and OS (HR: 4.2915, 95% CI: 1.7509–10.5182, P = 0.00145, Cox regression analysis, Table 1) between the PDE7B high expression group and PDE7B low expression group, it was showed that the PDE7B gene is an independent and unfavorable prognostic factor for CN-AML patients. The results also showed age (OS, HR: 2.8295, 95% CI: 1.3241–6.0462, P = 0.0072, Cox regression analysis, Table 1), DNMT3A (EFS, HR: 2.9058, 95% CI: 1.0958–4.3527, P = 0.0264, Cox regression analysis; OS, HR: 2.184, 95% CI: 1.3241–6.0462, P = 0.0072, Cox regression analysis, Table 1) and FLT3 (EFS, HR: 3.9216, 95% CI: 1.6126–9.5366, P = 0.0025, Cox regression analysis, Table 1) were also high risk factors for CN-AML, while NPM1 (EFS, HR: 0.376, 95% CI: 0.1426–0.9915, P = 0.0480, Cox regression analysis, Table 1) and NRAS (OS, HR: 0.1777, 95% CI: 0.0340–0.9282, P = 0.0405, Cox regression analysis, Table 1) may predict a better prognosis for CN-AML patients. BM-BLAST, WBC, PB-BLAST, TET2, IDH2, IDH1, RUNX1, WT1, CEBPA, PTPN11 and KRAS were not found to differ significantly in our study.

**Table 1:** Multivariate analysis of 75 CN-AML patients from TCGA database.

Variables	EFS				OS			
	HR	Lower 95%	Upper 95%	P-value	HR	Lower 95%	Upper 95%	P-value
Age (≥60 vs < 60 years)	1.9491	0.86873	4.3731	0.105521	2.8295	1.32415	6.0462	0.00726
BM_BLAST (≥70% vs < 70%)	0.6623	0.28926	1.5165	0.329684	0.9845	0.4791	2.0229	0.966
WBC (≥30 vs < 30 × 10^9^/L)	0.6929	0.28804	1.667	0.412806	0.5845	0.24665	1.3849	0.2224
PB_BLAST ( ≥ 50% vs < 50%)	1.5847	0.64828	3.874	0.312701	1.2206	0.52258	2.8508	0.64514
DNMT3A (Mutation vs WT)	2.9058	1.3461	6.2727	0.006588	2.184	1.0958	4.3527	0.02642
NPM1 (Mutation vs WT)	0.376	0.1426	0.9915	0.048011	1.0693	0.44441	2.5731	0.88103
TET2 (Mutation vs WT)	0.6149	0.2185	1.7306	0.357035	0.3426	0.10597	1.1079	0.07365
FLT3 (Mutation vs WT)	3.9216	1.6126	9.5366	0.002579	1.034	0.46958	2.2768	0.93385
IDH2 (Mutation vs WT)	0.8216	0.23668	2.852	0.756947	0.6124	0.19942	1.8804	0.39157
IDH1 (Mutation vs WT)	1.6219	0.38208	6.885	0.512071	0.8082	0.19519	3.3463	0.76893
RUNX1 (Mutation vs WT)	1.4647	0.36591	5.8627	0.589697	1.9098	0.55304	6.5949	0.30621
NRAS (Mutation vs WT)	0.3428	0.07088	1.6575	0.183007	0.1777	0.03403	0.9282	0.04053
WT1 (Mutation vs WT)	2.2794	0.53949	9.631	0.262454	1.3021	0.383	4.4268	0.67244
CEBPA (Mutation vs WT)	1.3322	0.37097	4.7838	0.660162	1.6233	0.4718	5.5851	0.44224
PTPN11 (Mutation vs WT)	0.942	0.22872	3.8795	0.934037	1.5812	0.47179	5.2992	0.45777
KRAS (Mutation vs WT)	4.1015	0.86645	19.415	0.075201	1.0834	0.25536	4.5967	0.91348
PDE7B (High vs Low)	4.8206	1.98156	11.7273	0.000525	4.2915	1.75095	10.5182	0.00145

EFS, event-free survival time (months); OS, overall survival time (months); HR, hazard ratio; CI, confidence interval. BM-BLAST, bone marrow blast; WBC, white blood cell; PB-BLAST, peripheral blood blast; PDE7B (Phosphodiesterase 7B). DNMT3A, NPM1, TET2, FLT3, IDH2, IDH1, RUNX1, NRAS, WT1, CEBPA, PTPN11 and KRAS: AML related genes. The data analysis was performed by Cox regression analysis.

## Discussion

In 2000, J. M. Hetman *et al*. first systematically described PDE7B and identified it as a member of the PDE7 family with high affinity and specificity for cAMP^23^. In 2008, it has been reported that the expression of PDE7B was increased in CLL cells^20^. Subsequently, they had found that the PDE7B mRNA level of CLL patients was 9 times higher than that of the normal control group, and the median time-to-treatment (TTT) was shortened by several years compared with the low level PDE7B mRNA^16^. And then it has been suggested that cAMP can inhibit the growth of various types of lymphoma (especially CLL), while PDE7 inhibitors (nonselective for PDE7A and PDE7B) can kill CLL cells by increasing cAMP concentration, which may be a useful treatment for such diseases^21^. cAMP is an important second messenger in cell signaling, and intracellular cAMP levels are critical for the maturation and differentiation of leukemia cells^22^. Moreover, it was also found that elevated levels of cAMP lead to arrest of G1 phase in acute promyelocytic leukemia cells^24^. Therefore, it is not difficult to speculate that PDE7B, as a specific hydrolase of cAMP, may play an important role in the development of leukemia cells by regulating the concentration of cAMP.

AML is a type of malignant hematological disease originating from hematopoietic progenitor cells, nearly half of which is CN-AML. And more and more mutant genes have been found to contribute to the diagnosis and prognosis prediction of CN-AML patients. In the present study, we calculated and compared the gene expression profiles of CN-AML patients in multiple datasets, followed by survival analysis and prognostic risk analysis. And we have demonstrated a correlation between PDE7B gene and AML. The correlation is shown in the following points. 1) PDE7B is an independent prognostic predictor of CN-AML patients. 2) high expression level of PDE7B predicts a shorter survival time in either CN-AML patients or AML patients who have received Allo-HSCT or chemotherapy. 3) PDE7B is high expressed in LSC positive group and CD34^+^/CD38^+^ group.

LSCs are a group of tumor stem cells that have the ability to self-renew and produce heterogeneous leukemia cell populations. LSCs are an important cause of leukemia recurrence after different treatment regimens. And numerous studies have shown that LSC may be a new therapeutic target for hematological tumors^25,26^. As showed in our results, we compared the difference in the expression level of PDE7B between the LSC positive group and the LSC negative group and found that it was higher in the LSC positive group. It is currently accepted that CD34^+^/CD38^−^ is a molecular phenotype of the LSC^27^. In addition, acute leukemia is partially composed of immature progenitor cells with undetermined lineage^25^, and CD34^+^/CD38^+^ is a surface marker of hematopoietic progenitor cells^28^. Our results showed that the expression level of PDE7B gene was significantly elevated in the CD34^+^/CD38^+^ group. CD34^−^/CD38^−^ identifies differentiated mature cells in which PDE7B expression is reduced. As mentioned earlier, PDE7B is a specific hydrolase for cAMP which is critical for leukemia cell differentiation. So that might explain why PDE7B is higher in the CD34^+^/CD38^+^ group and lower in the CD34^−^/CD38^−^ group. Therefore, we can speculate that high expression of PDE7B may lead to a decrease in intracellular cAMP levels, which in turn prevents leukemia cells from further differentiation and maturation. In short, high levels of PDE7B may favor LSCs and produce a poor prognosis for AML.

Although genetic alterations are an important part of the pathogenesis of AML, many of the mutant genes (such as DNMT3A, FLT3, CEBPA, NPM1, TET2, IDH1, IDH2 etc.) which have been identified in recent years have contributed a lot to it. It is especially important for the diagnosis, prognosis prediction and targeted therapy of CN-AML patients. In our Cox proportional hazards regression model, we have calculated many AML-associated mutant genes and found that DNMT3A and FLT3 are high risk factors for CN-AML patients (HR > 1, P < 0.05), while NPM1 and NRAS are favorable genes (HR < 1, P < 0.05), and the PDE7B gene is the most significant one (unfavorable gene) by comparing the P value. In addition, we analyzed the relationship between those mutant genes and the expression level of PDE7B, and found that the three states (wild type, mutantion, and unknown) of IDH1 gene are significant difference between the PDE7B high group and the PDE7B low group.

Althoug our study have demonstrated that high level of PDE7B gene indicates poor prognosis in CN-AML patients. The mechanism of action of PDE7B in CN-AML is not clear and we just speculated that it may play a role by affecting the concentration of cAMP in CN-AML. In addition, how the PDE7B gene regulates LSC is also unknown. In general, more in-depth and sophisticated research needs to be done in the next step.

In conclusion, we found in our study that PDE7B gene is an independent prognostic predictor of CN-AML, and that high expression level of PDE7B gene is an unfavorable prognostic factor for CN-AML. The high expression of PDE7B gene has a significant correlation with leukemia stem cells. Anyway, this study reflects the potential and prospects of the PDE7B gene in the prognosis, targeted therapy, and risk stratification of AML patients.

## Methods

### Data sources

By performing the survival analysis of each gene with the data of gene expression and EFS (and OS) from The Cancer Genome Atlas (TCGA) CN-AML samples, the P value and hazard ratio (HR) of each gene were calculated. The PDE7B gene was in the top position by ranking the P value of each gene. Subsequently, we further analyzed the relationship between PDE7B expression levels and survival time of CN-AML patients, including TCGA (75 CN-AML samples), GSE12417^29^ U133B (162 CN-AML samples), GSE71014^30^ (104 CN-AML samples), GSE22778^31^ GPL8653 (54 CN-AML samples) GSE22778, GPL10107 (34 CN-AML samples), and a recently published study (203 CN-AML samples, http://www.vizome.org/)^32^. To understand the relationship between the expression level of PDE7B and the prognosis of patients with treated AML, we collected 67 AML samples who had received allogeneic hematopoietic stem cell transplantation (Allo-HSCT) and 92 AML samples who had received chemotherapy from the database TCGA and compared their survival time between the high expression level of PDE7B and the low level PDE7B group. Event-free survival time (EFS) was defined as from registration to death (for any reason), disease progression or recurrence, or last contact. Overall survival time (OS) was defined as from registration to death (for any reason) or last contact. And the expression levels of PDE7B under four molecular groups in GSE76004^33^ (227 samples from 78 AML patients were divided into CD34^−^/CD38^−^ group, CD34^−^/CD38^+^ group, CD34^+^/CD38^−^ group and CD34^+^/CD38^+^ group) were compared. In addition, samples in this dataset were classified according to LSC positive (138 samples) or LSC negative (89 samples), and the difference in expression of PDE7B between the two groups was compared. The study complies with the Helsinki Declaration.

### Expression analysis of PDE7B gene

We calculated the gene expression with the robust multiarray averaging (RMA) algorithm in the GEO database and detected the gene expression with RPKM from the RNA-seq datas in the TCGA database. The value of PDE7B gene expression was log transformed (log2). We used maximally selected rank statistics algorithm from survminer package to divide the test samples in each dataset into PDE7B high expression group and PDE7B low expression group.

### Statistics

Statistical analysis used R software v3.1.3 (ggplot2 and survminer package). Measurement data between the two groups were analyzed by unpaired t test (such as the difference of PDE7B expression between LSC positive group and LSC negative group), and multiple groups were tested by ANOVA test (such as the difference of PDE7B expression in different molecular types). Fisher’s exact test was used to analyze the numeration data (such as the deference of some baseline features of AML between high expression level of PDE7B and low level PDE7B). Kaplan-Meier survival curves and Log-rank test were used for survival analysis in AML patients. The Cox proportional hazards regression model for multivariate analysis was used to analyze the prognosis of multivariates associated with CN-AML. In any case, P < 0.05 was considered statistically significant.

## Supplementary information

Supplementary data
