# Supplementary material for: The Prognostic Significance of PDE7B in Cytogenetically Normal Acute Myeloid Leukemia
Source: Sci Rep. 2019 Nov 18;9:16991. doi: 10.1038/s41598-019-53563-x (PMC6861270; doi:10.1038/s41598-019-53563-x)

The Prognostic Significance of PDE7B in cytogenetically normal Acute Myeloid Leukemia

Ling Cao[1*], Weilong Zhang[2*], Xiaoni Liu[3*], Ping Yang[2], Jing Wang[2], Kai Hu[2], Xiuru Zhang[4], Weiyou Liu[3], Xue He[4★], Hongmei Jing[2★], Xiaoliang Yuan[3★]

Supplementary Table 1. Baseline characteristics of 75 CN-AML patients in the TCGA database according to the level of PDE7B.

Characteristics	Level	PDE7B-low	PDE7B-high	P-value
n		35	40	
Sex (%)	Female	19 (54.3)	19 (47.5)	0.646
	Male	16 (45.7)	21 (52.5)	
Race (%)	Black	0 (0.0)	3 (7.5)	0.353
	Others	0 (0.0)	1 (2.5)	
	Unknow	6 (17.1)	7 (17.5)	
	White	29 (82.9)	29 (72.5)	
FAB (%)	M0	1 (2.9)	2 (5.0)	0.541
	M1	13 (37.1)	11 (27.5)	
	M2	9 (25.7)	10 (25.0)	
	M4	8 (22.9)	7 (17.5)	
	M5	3 (8.6)	9 (22.5)	
	M7	0 (0.0)	1 (2.5)	
	Unknow	1 (2.9)	0 (0.0)	

Age (mean (sd))		51.37 (18.38)	57.35 (15.51)	0.131
BM_BLAST (mean (sd))		70.91 (18.80)	70.58 (18.20)	0.937
WBC (mean (sd))		32.95 (55.53)	67.00 (52.01)	0.008
PB_BLAST (mean (sd))		41.54 (33.80)	47.27 (33.14)	0.461
Karyotype (%)	Normal	35 (100.0)	40 (100.0)	NA
Risk (%)	Intermediate	35 (100.0)	40 (100.0)	NA
Induction (%)	7+3	17 (48.6)	14 (35.0)	0.475
	7+3+3	8 (22.9)	11 (27.5)	
	7+3+3+others	0 (0.0)	3 (7.5)	
	7+3+others	5 (14.3)	3 (7.5)	
	Cytarabine	0 (0.0)	1 (2.5)	
	Decitabine	3 (8.6)	2 (5.0)	
	Decitabine+others	1 (2.9)	0 (0.0)	
	Hydrea+others	0 (0.0)	1 (2.5)	
	no treatment	0 (0.0)	1 (2.5)	
	Others	0 (0.0)	1 (2.5)	
	Revlimid	1 (2.9)	3 (7.5)	
Transplant (%)	Auto	3 (8.6)	1 (2.5)	0.717
	Chemotherapy	16 (45.7)	21 (52.5)	
	Haplo	1 (2.9)	0 (0.0)	
	MUD	8 (22.9)	8 (20.0)	
	no treatment	0 (0.0)	1 (2.5)	

	sib Allo	7 (20.0)	9 (22.5)	
Before_transplant (%)	CR 1	10 (28.6)	8 (20.0)	0.719
	CR 2	4 (11.4)	3 (7.5)	
	CR 3	0 (0.0)	1 (2.5)	
	No transplant	16 (45.7)	21 (52.5)	
	no treatment	0 (0.0)	1 (2.5)	
	Others	4 (11.4)	4 (10.0)	
	Rel 1	0 (0.0)	2 (5.0)	
	Rel 2	1 (2.9)	0 (0.0)	
Relapse (%)	No	15 (42.9)	13 (32.5)	0.473
	Yes	20 (57.1)	27 (67.5)	
DNMT3A (%)	WT	25 (71.4)	20 (50.0)	0.097
	Mutation	10 (28.6)	19 (47.5)	
	Unknow	0 (0.0)	1 (2.5)	
NPM1 (%)	WT	18 (51.4)	13 (32.5)	0.158
	Mutation	17 (48.6)	26 (65.0)	
	Unknow	0 (0.0)	1 (2.5)	
TET2 (%)	WT	29 (82.9)	36 (90.0)	0.289
	Mutation	6 (17.1)	3 (7.5)	
	Unknow	0 (0.0)	1 (2.5)	
FLT3 (%)	WT	25 (71.4)	19 (47.5)	0.059
	Mutation	10 (28.6)	20 (50.0)	

	Unknow	0 (0.0)	1 (2.5)	
IDH2 (%)	WT	29 (82.9)	35 (87.5)	0.5
	Mutation	6 (17.1)	4 (10.0)	
	Unknow	0 (0.0)	1 (2.5)	
IDH1 (%)	WT	27 (77.1)	38 (95.0)	0.01
	Mutation	8 (22.9)	1 (2.5)	
	Unknow	0 (0.0)	1 (2.5)	
RUNX1 (%)	WT	32 (91.4)	35 (87.5)	1
	Mutation	3 (8.6)	4 (10.0)	
	Unknow	0 (0.0)	1 (2.5)	
NRAS (%)	WT	33 (94.3)	35 (87.5)	0.679
	Mutation	2 (5.7)	4 (10.0)	
	Unknow	0 (0.0)	1 (2.5)	
WT1 (%)	WT	32 (91.4)	37 (92.5)	0.82
	Mutation	3 (8.6)	2 (5.0)	
	Unknow	0 (0.0)	1 (2.5)	
CEBPA (%)	WT	31 (88.6)	35 (87.5)	1
	Mutation	4 (11.4)	4 (10.0)	
	Unknow	0 (0.0)	1 (2.5)	
PTPN11 (%)	WT	32 (91.4)	37 (92.5)	0.82
	Mutation	3 (8.6)	2 (5.0)	
	Unknow	0 (0.0)	1 (2.5)	

KRAS (%)	WT	34 (97.1)	37 (92.5)	1
	Mutation	1 (2.9)	2 (5.0)	
	Unknow	0 (0.0)	1 (2.5)	

FAB, French–American–British subtypes; MUD, matched unrelated donor; sib-allo HSCT, sib allogeneic hematopoietic stem cell transplantation; Haplo, haploidentical; CR 1, first complete response; Rel 1, first relapse; WT, wild type. Measurement data were compared by unpaired t test (two sided). The comparison of the enumeration data was performed by Fisher's exact test.

Supplementary figure legends

Supplementary Figure 1. The expression level of the PDE7B gene in 75 CN-AML patients from the TCGA database. The unpaired t test (two sided) is used for comparison between the two groups. The x-axis represents PDE7B high/low and the y-axis represents gene expression level of PDE7B (log2).

Supplementary Figure 2. The expression level of the PDE7B gene in samples from the GEO and TCGA database. A. The expression levels of PDE7B in the datasets GSE12417 U133B (left) and GSE71014 (right) are shown. B. The expression levels of PDE7B in the datasets GSE22778 GPL8653 (left) and GSE22778 GPL10107 (right) are shown. C. Represents the expression level of PDE7B in 67 AML patients who had received Allo-HSCT (left) and 92 AML patients who had received chemotherapy (right) from the TCGA database. The unpaired t test (two sided) is used for comparison between the two groups. The x-axis represents PDE7B high/low and the y-axis represents gene expression level of PDE7B (log2).

Supplementary Figure 3. Comparison of the OS time between high PDE7B group and low PDE7B group in patients from Vizome dataset. A. The OS time (days) of 396 AML patients (left) and 203 CN-AML patients (right) between high level PDE7B and low level PDE7B are compared. The survival curve is compared with the log-rank test. The x-axis represents the survival time and the y-axis represents the survival probability. B. On the left are PDE7B expression levels of 396 AML patients and on the right are 203 CN-AML patients. The unpaired t test (two sided) is used for comparison between the two groups. The x-axis represents PDE7B high/low and the y-axis represents gene expression level of PDE7B.

Supplementary Figure 1

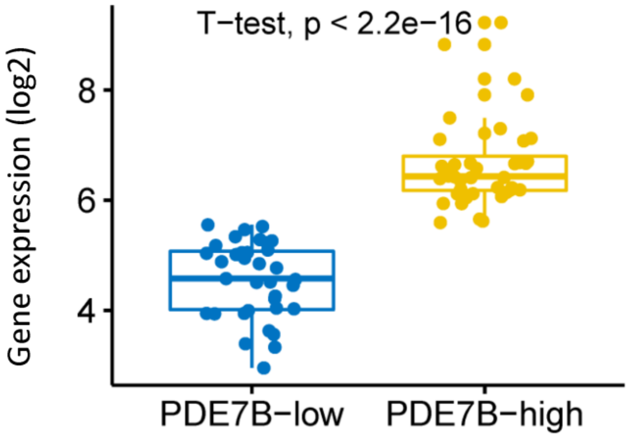

Supplementary Figure 2

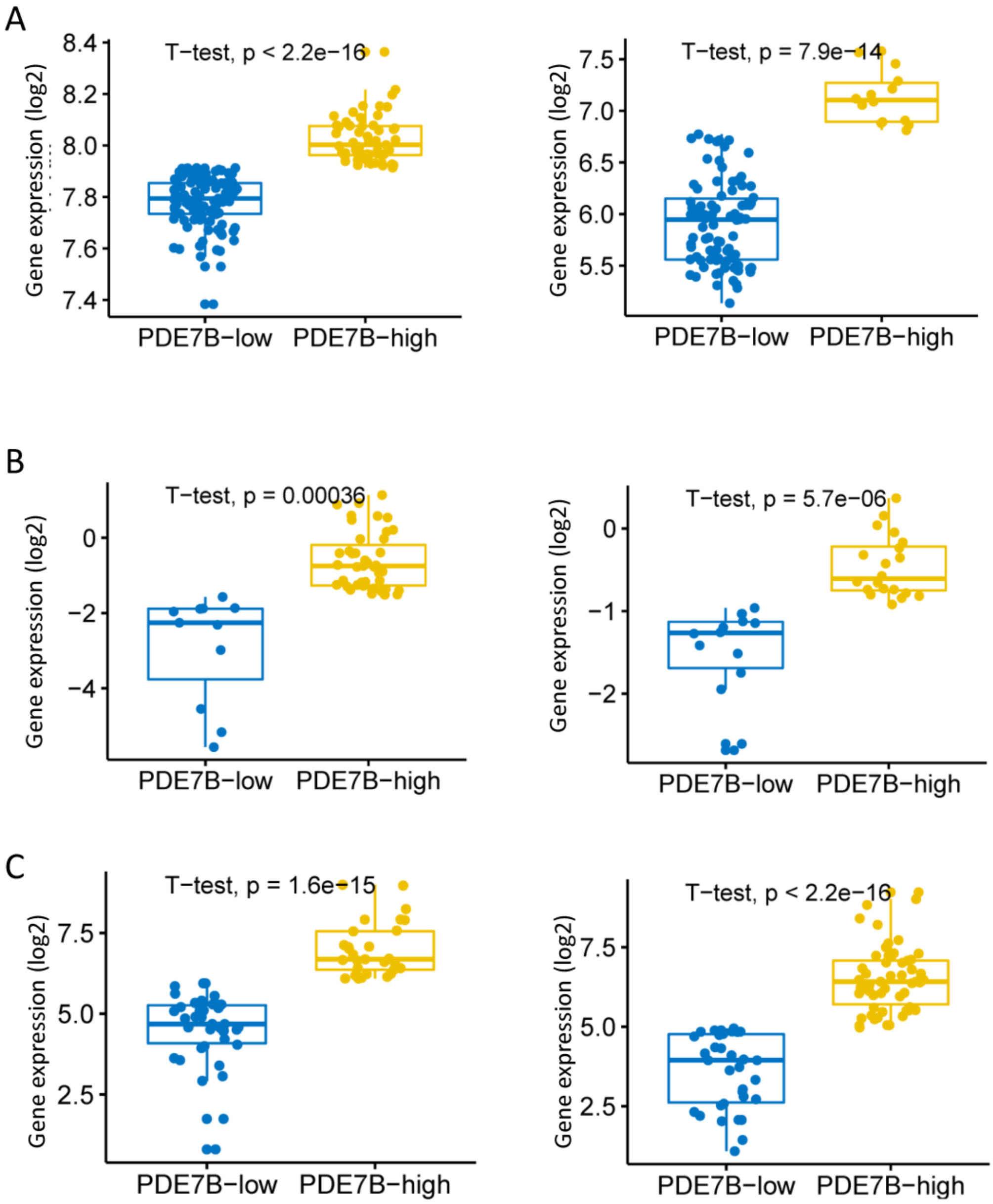

Supplementary Figure 3

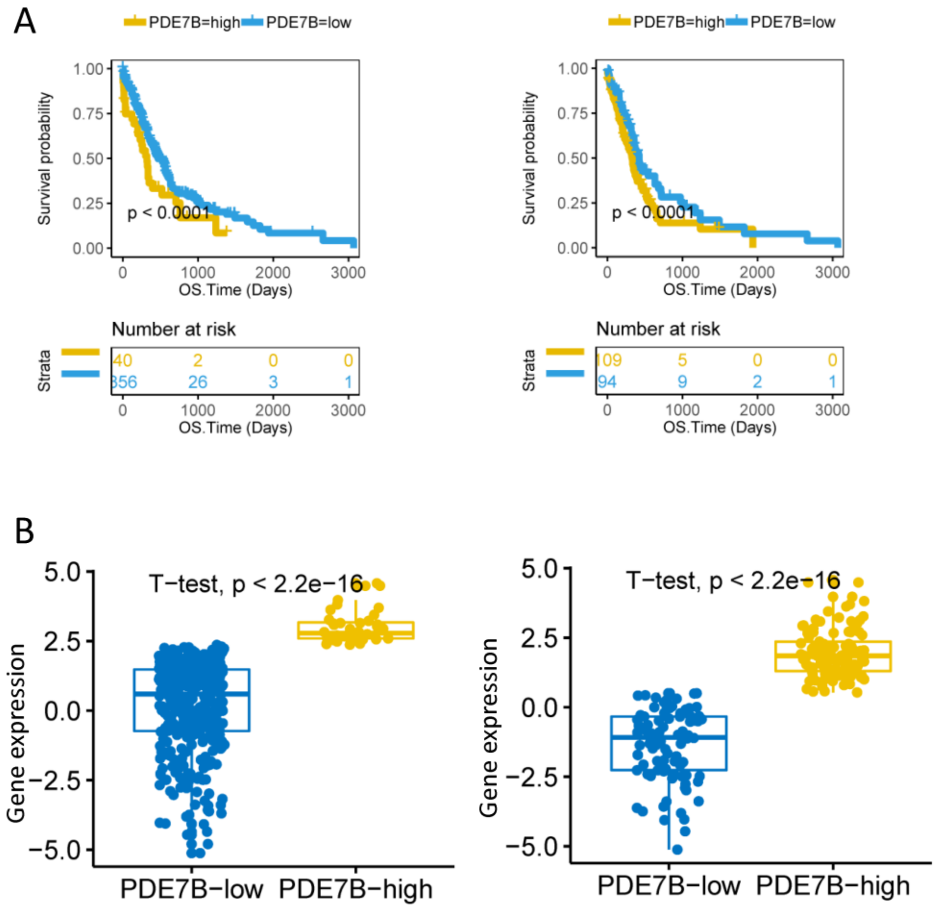

Supplement: Supplementary file 1 — Supplementary data [file 41598_2019_53563_MOESM1_ESM.pdf]
